# Medial Tibial Stress Syndrome: A Review Article

**DOI:** 10.7759/cureus.26641

**Published:** 2022-07-07

**Authors:** Nikita S Deshmukh, Pratik Phansopkar

**Affiliations:** 1 Musculoskeletal Physiotherapy, Ravi Nair Physiotherapy College, Datta Meghe Institute of Medical Sciences, Wardha, IND

**Keywords:** conservative management, exertional leg pain, medial tibial stress syndrome, runners, shin pain

## Abstract

Leg pain caused by recurrent stressors is known as shin pain, also known as the medial tibial stress syndrome (MTSS). Athletes, particularly runners, are more vulnerable. As a result, runners devote little time to practice and avoid exercises completely. The precise cause is yet to be identified. Microdamage caused by recurrent stressors has been proposed as the fundamental mechanism in other investigations. Gender, navicular bone loss, higher body mass index, activities of high intensity, and increased range in external hip rotation in males are all risk factors. A common complaint is a bilateral pain in the distal leg, primarily on the anterior and medial sides. Pain is exacerbated by activity and eased by relaxation. Particularly, pain and swelling in the posterior and medial aspects of the tibia, as well as other causative symptoms, may be discovered during the examination. To rule out alternate origins of the same symptoms, imaging modalities such as computed tomography, radiography, bone scintigraphy, and magnetic resonance imaging might be used. Preventative measures include shock-absorbing insoles, repetitive stress avoidance, and effective treatment of repetitive stress disorder and anatomical abnormalities. Rest, ice, and pain medications are the most common treatments.

## Introduction and background

Medial tibial stress syndrome (MTSS) is the discomfort and pain in the leg region due to repetitive pressure. It is one of the most common overuse issues in runners and the community, affecting almost 35% of the athletic population. Overuse injuries like MTSS can impact up to 70% of runners in a year [1]. Running and other sports like American football and basketball have been demonstrated to have a high MTSS. Overuse injuries, such as shin splints, account for 18.5% of all soccer-related disabilities [2]. In several groups, exercise-related leg pain is the common cause of MTSS, but the exact reason is unknown. As soon as feasible, MTSS must be identified and handled. Otherwise, restrictions develop as a result of frequent shocks [1]. As recreational running and jogging have grown in popularity, so has the number of ailments classed as overuse injuries. Shin splints are the most prevalent exercise-related injuries. Because of the vagueness of the term “shin splint”, appropriate diagnosis, treatment, and prevention can be challenging. According to research, exercise-induced shin discomfort is responsible for 10-20% of all runner injuries and 60% of all lower-limb overuse injuries [3]. According to military studies, shin splints are also the most common injury among ballet dancers, with 4-10% of recruits diagnosed with them throughout their eight to 12 weeks of basic training [4].

## Review

Definition

In leg runners, inflammatory traction most commonly occurs in the tibial region. "It is a more elaborated nomenclature which simply explains the medial tibial periostitis or tibial traction of medial periostitis" [5,6].

Location

Shin splints do not usually manifest themselves in the same location. According to a study, shin splint pain is more common in the distal third of the tibia on the medial side or in the proximal region, as well as the anterior, posterior, lateral, or medial side of the leg. The research investigated chronic medial shin pain, while others investigated chronic anterolateral shin pain, and some investigated both sides [7,8].

Pathogenesis and risk factors

Repetitive stress that generates microdamage beyond the repair threshold could be a mechanism for developing MTSS. Various types of bone stress differ from the appropriate stress of enough rest, which strengthens it [7]. When bending pressure on the tibia is more significant than the strength of opposite leg muscles, it is considered MTSS. Periostitis and repetitive tibial bending and bowing have been associated with MTSS. Periostitis can be caused by several causes, including exercise intensity and training surfaces. Furthermore, a sudden increase in training volume on hard surfaces is a training error, and ageing footwear is the commonest cause of MTSS. According to the literature, there is a link between the deep posterior compartment fascia and the MTSS [9]. Because periosteal traction can be caused by the soleus and tibialis posterior muscles, they are assumed to be implicated. It is also been hypothesized that flexor digitorum longus contractions put extra strain on the tibial fascia. Periosteum muscle traction can induce MTSS, with the soleus being the most common [8].

Clinical presentation

Bilateral soreness or pain in the medial side of the tibia, more commonly in the distal region, is common in people with MTSS. A common source of pain is the central section of the medial tibia. On the other hand, shin splints can affect the entire length of the leg [10]. It is usual to describe lateral side soreness as dull and unpleasant. On movement, pain increases, and at rest, pain is subsided; it most commonly occurs at the initiation of a workout and lessens as the workout continues [6]. The pain is usually more substantial the following day, but it will go away gradually. Pain can be felt even while the patient rests in severe and persistent MTSS. Radiation to the foot, as well as dysesthesia, has been observed. Stress fractures and other overuse injuries have comparable indications and symptoms [7]. As a result, maintaining a high level of suspicion is critical. The doctor should start the examination by checking for indicators of infection, such as wearing sole-free shoes or like limb length discrepancy abnormality [11]. MTSS shows other additional features, such as pain while the percussion is administered and discomfort while hopping; it has been demonstrated that posteromedial border soreness of the tibia is the most sensitive [12]. Mild oedema with subcutaneous thickening of the tibial line can also be seen in MTSS patients. It is important to distinguish this from a visible callus [1]. Passive stretching may disclose weakness and soreness throughout the assessment. Stress fractures are more likely to cause localized pain, discomfort, and oedema. Hence, they must be ruled out clinically. In addition, while evaluating persons with MTSS, disorders such as tendinitis, compartment syndrome, popliteal artery compression, vasculitis, and nerve impingement are also considered [9].

Incidence

Of the population, females (55.3%) were more likely to use a splint than males (44.7%). Shin splints are more common among marathon runners, depending on the duration of pain and the shoe surface [10].

Cause

MTSS commonly occurs in runners and jumpers who overexert themselves or go too quickly for their abilities. Sudden progression in the exercise program, such as an increase in length and intensity, could be associated with this condition [6]. Running on uneven or hard surfaces and wearing unsuitable running shoes (with limited shock absorption) could play a role in the disaster [10]. Among the most typically reported intrinsic variables are biomechanical anomalies such as foot arch deformities, excessive pronation of the foot, and uneven leg length [13]. Women are more prone than men to have stress fractures, especially if they already have one. Dietary, biomechanical, and hormonal variables influence this. People who are obese are more likely to have this condition [11]. As a result, overweight individuals should take a healthy diet combined with exercise or should attempt to lose weight before therapy begins or start an organized training program [13]. These people, as well as those who are under-trained, should work on improving their fitness over time. Because cold weather aggravates this condition, it is much more necessary (than usual) to warm up properly [14].

Investigation

Imaging modality rules out a proper investigation, but it is still in question. However, imaging is frequently needed to rule out alternative possibilities like a stress fracture or chronic exertional compartment syndrome [15]. Radiographs, computed tomography (CT), magnetic resonance imaging (MRI), and bone scintigraphy are among the imaging modalities available. Even though the fracture may not be visible for two weeks after callus formation, radiography is frequently utilized to rule out stress fractures [14]. A posteromedial periosteal reaction may also be evident. However, it is less obvious than in other imaging modalities. According to Gaeta et al., all long-distance runners showed CT scan abnormalities [12]. The results of a CT scan are divided into three categories. Type 0 appeared to be completely healthy. In the absence of osteopenia, type 1 has a dispersed and somewhat reduced cortical attenuation. Cavitation or striations describe type 2 osteopenia [16]. With a sensitivity rate of 88%, MTSS MRI is the most sensitive investigation to rule out soft tissue involvement. The most common MRI finding is unilateral bone marrow oedema, followed by periosteal oedema (35%). A regular MRI scan is also possible for patients with clinical MTSS [15]. The good correlation between chronicity and normal pictures is a drawback of MRI in MTSS. MTSS biopsy infiltration by chronic inflammatory cells and fibrous thickening is more prevalent. Histology, on the other hand, is inconclusive in MTSS [10]. The issue of pressure studies has been extensively investigated in the past. Neural feelings, anterior tibial discomfort, pain that persists after activity, cramp-like pain, and other symptoms that do not go away after rest are all common symptoms that may indicate the need for pressure tests [3].

Prevention

"Overstress avoidance" has been coined as a method of preventing MTSS. This is critical because repetitive injuries can occur before symptoms arise. According to some estimates, training errors are at fault for 50% of all running-related injuries [17]. These factors contribute to physical exhaustion and obstruct the body's natural stress responses. Warm-up for 10-15 minutes and then stretching exercises, using proper sole footwear, and progressive training exercises can all help prevent injuries, but their effectiveness is debatable and based on little evidence [18]. Shock-absorbent insoles and other shoe adjustments can help avoid MTSS. Insoles controlling pronation are also advantageous, particularly for persons with a navicular drop [8].

Treatment

The most commonly utilized treatment of shin splints is currently supported by limited evidence in the literature. Treatments include cryotherapy, stretching and strengthening exercises, nonsteroidal anti-inflammatory drugs, and adjustments in training programs, which vary according to an individual. Orthotic devices are used to correct biomechanical anomalies, and the rest are all traditional treatments [11].

Cryotherapy

Compared to other treatments such as instrument-assisted soft tissue mobilization and myofascial release, patients with shin splints allocated to a therapy program consisting primarily of rest and ice application achieved statistically significant effects. Ice massage or crushed ice is widely used in military institutions to treat lower-extremity overuse ailments like shin splints [10].

Stretching and Strengthening

For the treatment of shin splints, stretching is widely used, but new research suggests it is ineffective. Most commonly prescribed treatments for shin splints are triceps surae complex stretches four to five times, calf stretch in sitting and standing position, as sitting with a towel helps to stretch the calf, and ankle-strengthening activities [13]. The efficacy of shin splint stretching and strengthening exercises in treating shin splints is still up for dispute [14]. Modification of training regimens investigates the impact of changing training aspects such as frequency and duration, running distance reduction, and graduated running on injury incidence. Reduced training frequency, length, and total running distance were found to impact injury reduction substantially [6]. Soft tissue injuries may be reduced by reducing the reach, frequency, and duration of running. The most common treatment for MTSS is steroid injections. Several studies have demonstrated that rest from high-impact exercise is an effective therapy option for overuse injuries [19]. On the other hand, sources could not agree on a specific amount of rest or when it should be taken. It is usually not a good idea to resume normal activities until the soreness has completely disappeared. A gradual return to the entire exercise is crucial in preventing shin splints from reoccurring.

Shoe Orthotics

Shock-absorbing insoles could cut the number of stress fractures in half. The positive treatment outcome should not be underestimated, despite the low to intermediate quality data [12].

The treatment goal for pain relief

Cold application on the area affected with the rest of the affected region has demonstrated an efficient therapeutic strategy in the acute stage. Analgesia can be provided with acetaminophen and nonsteroidal anti-inflammatory medications [7]. Calf muscle stretching, planter stretching, and strengthening calf muscle activities have been used in patients with MTSS in physical therapy. Extracorporeal shockwave therapy, on the other hand, may be helpful [11]. Once the pain has subsided, the focus of treatment should shift to workout changes. This can be accomplished by reducing training intensity, activity frequency, and running distance [16].

Acute Phase

Rest improves symptoms, and rest with medication for two to six weeks is most commonly effective for symptoms and early recovery. Acetaminophen and anti-inflammatory drugs are often used. Analgesic gels may be used after 20 minutes of activity. Also, cryotherapy or an ice pack is usually used to relieve pain [15]. While many physical therapeutic modalities, including instrument-assisted soft tissue mobilization on fascial release, ultrasound therapy, and electrical stimulation can be employed in the acute period, there is no evidence that they are effective [13].

Subacute Phase

The main goal of treatment is to improve the training condition or biomechanical concern due to the condition. Running distance, intensity, and frequency could be cut in half if training settings were changed. It is suggested to prevent walking on uneven surfaces and avoid rough terrain. Low-impact activities must be done during the rehabilitation phase. Also, cross-training exercises are recommended, such as jogging and aquatic therapy [10]. The athlete must start with the low impact training, which gradually progresses to hill running with increased speed, intensity, and distance. The activity starts after a week when the pain subsides. To avoid muscle fatigue, add a rehabilitation program with stretching and strengthening of calf muscle exercise. Core hip muscle strengthening also benefits the patient [4]. Developing strong core, hip, and gluteal muscles for core stability improves running activity with proper mechanics and avoids overuse of lower extremities. Neuromuscular education such as balancing or proprioceptive balance training is also helpful. This can be done with the help of a balance board or single-leg standing [9]. To further prevent injury, balance activities, improving running mechanics, and improving muscle stabilization with strengthening and stretching exercises are recommended. Proper shoes with appropriate soles will help proper shock absorption, preventing re-injury.

For the biomechanical problem of feet, an orthosis can also be used, which also helps people with further management. Also, for the avoidance of excessive foot pronation and pes planus (flexible/semi-rigid), an orthosis can help. In extreme cases, a pneumatic brace or cast is required sometimes [11]. Manual therapy can be used for various biomechanical disorders of the spine, sacroiliac joint, and muscle imbalances. They are usually used to keep an existing injury from recurring [6].

## Conclusions

MTSS is the most prevalent condition that occurs commonly in runners between 20 and 30 years of age. Pain in the shin area affects the anteroposterior aspect of the tibia as well as the posterolateral aspect of the leg. Many clinical investigations are available or assessable to find out the condition. MRI and CT scans can find the exact finding of the condition. Prevention is the first-line treatment and it helps prevent the worsening of the injury. Many studies suggest conservative treatment for MTSS and relief from generalized symptoms, but in this article, we explain the conservative management options like cryotherapy, stretching and strengthening, and shoe orthosis, as well as the recent advanced therapy options like extracorporeal shockwave therapy, to help relieve symptoms. This review article aims to represent the effective treatment for MTSS, which helps reduce pain and improve functions in a short period of time to continue the activity as it is. This article would be helpful for future studies in the same condition and it provides a brief review of the condition to help clinical practitioners.

## References

[1] Bates P (1985). Shin splints--a literature review. Br J Sports Med.

[2] Alfayez SM, Ahmed ML, Alomar AZ (2017). A review article of medial tibial stress syndrome. J Musculoskelet Surg Res.

[3] Wilder RP, Sethi S (2004). Overuse injuries: tendinopathies, stress fractures, compartment syndrome, and shin splints. Clin Sports Med.

[4] Ross J (1993). A review of lower limb overuse injuries during basic military training. Part 1: types of overuse injuries. Mil Med.

[5] Patel PY, Patil N (2020). Prevalence of shin splint in recreational marathon runner. Int J Physiother.

[6] Pietrzak M (2014). Diagnosis and management of acute medial tibial stress syndrome in a 15 year old female surf life-saving competitor. Int J Sports Phys Ther.

[7] DeLacerda FG (1980). A study of anatomical factors involved in shinsplints. J Orthop Sports Phys Ther.

[8] Johnell O, Rausing A, Wendeberg B, Westlin N (1982). Morphological bone changes in shin splints. Clin Orthop Relat Res.

[9] Finch PM (1998). Chronic shin splints: a review of the deep posterior compartment. Foot.

[10] Thacker SB, Gilchrist J, Stroup DF, Kimsey CD (2002). The prevention of shin splints in sports: a systematic review of literature. Med Sci Sports Exerc.

[11] Fick DS, Albright JP, Murray BP (1992). Relieving painful 'shin splints'. Phys Sportsmed.

[12] Johnston E, Flynn T, Bean M, Breton M, Scherer M, Dreitzler G, Thomas D (2006). A randomized controlled trial of a leg orthosis versus traditional treatment for soldiers with shin splints: a pilot study. Mil Med.

[13] Bhandakkar PA, Naqvi W (2020). Impact of physiotherapy rehabilitation on patients with bilateral osteoarthritis knee pain--a case report. J Evol Med Dent Sci.

[14] Athawale V, Phansopkar P, Darda P, Chitale N, Chinewar A (2022). Impact of physical therapy on pain and function in a patient with scoliosis. Cureus.

[15] Couture CJ, Karlson KA (2002). Tibial stress injuries: decisive diagnosis and treatment of 'shin splints'. Phys Sportsmed.

[16] Reinking MF (2007). Exercise related leg pain (ERLP): a review of the literature. N Am J Sports Phys Ther.

[17] Pawar A, Pathan H, Rao R, Mishra G (2021). A case report of fractured tibia with improved outcomes through early physiotherapy approach. Biosci Biotechnol Res Commun.

[18] Thacker SB, Gilchrist J (2004). The Impact of Stretching on Sports Injury Risk: A Systematic Review of the Literature. Med Sci Sports Exerc.

[19] Bele AW, Qureshi MI, Dhankar S (2020). Impact of fall on anterior cruciate ligament of 33-year-old male. J Datta Meghe Inst Med Sci Univ.

